# Odon device for instrumental vaginal deliveries: results of a medical device pilot clinical study

**DOI:** 10.1186/s12978-018-0485-8

**Published:** 2018-03-12

**Authors:** Javier A. Schvartzman, Hugo Krupitzki, Mario Merialdi, Ana Pilar Betrán, Jennifer Requejo, My Huong Nguyen, Effy Vayena, Angel E. Fiorillo, Enrique C. Gadow, Francisco M. Vizcaino, Felicitas von Petery, Victoria Marroquin, María Luisa Cafferata, Agustina Mazzoni, Valerie Vannevel, Robert C. Pattinson, A Metin Gülmezoglu, Fernando Althabe, Mercedes Bonet, for the World Health Organization Odon Device Research Group, José Belizán, Eduardo Bergel, Franco Borruto, Alain Treisser, Michel Boulvain, Gian Carlo Di Renzo, Justus Hofmeyr, Kevin Judge, Tak Yeung Leung, Ola Didrik Saugstad, Ana Pilar Betrán, Mario Merialdi, Jennifer Requejo, Marleen Temmerman, Hugo Krupitzki, Javier A. Schvartzman, Fernando Althabe, Maria Luisa Cafferata, Roberto Casale, Lucio Rivola, Silvana Varela, Gerardo Murga, Elena Hurtado, Alfred Osoti, Zahida Qureshi, Dalene Barnard, Robert Pattinson, Valerie Vannevel, Salome Maswime, Hennie Lombaard, Stefan Gebhardt, Sabaratnam Arulkumaran, Khalid Khan, Nina Kimmich, Tina Lavender, Pisake Lumbiganon, Nafissa Osman, Effy Vayena, Gijs Walraven, Diane Whitham, Khaled Yunis, Mercedes Bonet, A. Metin Gülmezoglu, Ndema Abu Habib, My Huong Nguyen

**Affiliations:** 1 Department of Obstetrics and Gynecology, Centro de Educación Médica e Investigaciones Clínicas “Norberto Quirno” (CEMIC-IUC - CONICET), University Hospital, Av. Galván 4102 1431FWO, Buenos Aires, Argentina; 2 UNDP/UNFPA/UNICEF/WHO/World Bank Special Programme of Research, Development and Research Training in Human Reproduction (HRP), Department of Reproductive Health and Research, World Health Organization, Avenue Appia 20, CH-1211 Geneva 27, Switzerland; 3 Becton Dickinson and Company (BD), Franklin Lakes, NJ USA; 4 Partnership for Maternal, Newborn and Child Health, World Health Organization, Avenue Appia 20, CH-1211 Geneva 27, Switzerland; 5 Department of Health Sciences and Technology, ETH Zurich, Auf der Mauer 17, 8092 Zurich, Switzerland; 6 Instituto de Efectividad Clínica y Sanitaria (IECS - CONICET), Dr Emilio Ravignani 2024, C1414CPV Buenos Aires, Argentina; 7 SAMRC Maternal and Infant Health Care Strategies, Department of Obstetrics and Gynaecology, University of Pretoria, Pretoria, South Africa

**Keywords:** Instrumental vaginal delivery, Odon device, Second stage of labour

## Abstract

**Background:**

A prolonged and complicated second stage of labour is associated with serious perinatal complications. The Odon device is an innovation intended to perform instrumental vaginal delivery presently under development. We present an evaluation of the feasibility and safety of delivery with early prototypes of this device from an early terminated clinical study.

**Methods:**

Hospital-based, multi-phased, open-label, pilot clinical study with no control group in tertiary hospitals in Argentina and South Africa. Multiparous and nulliparous women, with uncomplicated singleton pregnancies, were enrolled during the third trimester of pregnancy. Delivery with Odon device was attempted under non-emergency conditions during the second stage of labour. The feasibility outcome was delivery with the Odon device defined as successful expulsion of the fetal head after one-time application of the device.

**Results:**

Of the 49 women enrolled, the Odon device was inserted successfully in 46 (93%), and successful Odon device delivery as defined above was achieved in 35 (71%) women. Vaginal, first and second degree perineal tears occurred in 29 (59%) women. Four women had cervical tears. No third or fourth degree perineal tears were observed. All neonates were born alive and vigorous. No adverse maternal or infant outcomes were observed at 6-weeks follow-up for all dyads, and at 1 year for the first 30 dyads.

**Conclusions:**

Delivery using the Odon device is feasible. Observed genital tears could be due to the device or the process of delivery and assessment bias. Evaluating the effectiveness and safety of the further developed prototype of the BD Odon Device™ will require a randomized-controlled trial.

**Trial registration:**

ANZCTR ACTRN12613000141741 Registered 06 February 2013. Retrospectively registered.

## Plain English summary

The Odon device is an innovation, presently under development, intended to assist vaginal birth when second stage takes longer than what is considered safe or if complications arose (e.g. baby is large or distressed). The objective of the study was to find out whether this new device helps pushing out of the baby through the birth canal. The study included women at their first delivery and women who delivered before, with uncomplicated pregnancies and one fetus in two hospitals in Argentina and South Africa. Delivery with the Odon device was attempted in women undergoing normal, uncomplicated labour. The Odon device was inserted successfully in 46 of the 49 women included (93%), and successful delivery with expulsion of the fetal head after one-time application of the Odon device was achieved in 35 (71%) women. Genital tears occurred in 29 (59%) women. As the use in humans has been limited, increased risk of tears and other unknown risks cannot be ruled out. Four women had cervical tears but no women had severe perineal trauma. All babies were born alive and vigorous. No adverse maternal or infant outcomes were observed at 6-weeks follow-up, and at 1 year for the first 30 mothers and babies. Delivery using the Odon device is feasible. These findings suggest continuing evaluating the effectiveness and safety of new prototypes of the BD Odon Device™ in a clinical comparative trial with a standard device before introduction in clinical practice.

## Background

Prolonged or complicated second stage of labour is associated with potentially serious maternal complications and deaths as well as stillbirths and neonatal morbidity and mortality [1].

Currently, the main options for managing prolonged/complicated second stage of labour are instrumental vaginal delivery (IVD) with forceps or vacuum extractor, and caesarean section. IVD is one of the six critical functions of basic emergency obstetric and neonatal care [2], but currently under-used, particularly in low-resource settings where rates are as low as 1-5% [3, 4]. In high-resource settings, IVD rates tend to be higher (up to 15%) [5, 6], but declining rates have been reported in several countries [4]. These trends are inversely correlated with the increasing rates of caesarean sections worldwide [7].

There are multiple factors associated with low or declining use of IVD. None of the available instruments are without risk for the mother or the baby. While use of forceps is associated with increased maternal perineal trauma, need for analgesia and neonatal facial injury, cephalhaematoma and subgaleal haemorrhage are associated with vacuum birth [8]. Failure rates are also reported to be relatively high, particularly with the use of vacuum extractor at around 20% [8]. An additional barrier is the high level of skill and continuous training required to perform safe and effective IVD [9, 10]. This limits the use of IVD if birth attendants are not provided with sufficient resources to obtain and maintain the necessary skills.

The design and development of innovative IVD instruments that are safe for mothers and babies, easy for different cadres of skilled birth attendants to use, cost-effective, and affordable in low resource settings is a priority [11]. In this sense, the Odon device is a technological innovation intended to fulfil this gap, by improving outcomes associated with prolonged or complicated second stage of labour and reduce the skill level and equipment required to perform assisted vaginal deliveries.

We present results of an early terminated study designed to evaluate the feasibility and preliminary safety of delivery with early prototypes of the Odon device in singleton term pregnancies under non-emergency conditions.

## Methods

### Study design and participants

This was a hospital-based, multi-phased, open-label, medical device pilot clinical study without control group. The study methods were described in detail elsewhere [12].

The design of the study in phases included an evaluation of the first five multiparous women, the next 25 multiparous with 1 year follow-up, and then the inclusion of both multiparous and nulliparous women until completion of the sample size. Therefore, women were enrolled in three phases: 1) multiparous women with a previously successful spontaneous vaginal delivery with 1 year follow up (2011-2012); 2) multiparous and nulliparous with 6-weeks follow up (2014-2015) at a private not-for-profit tertiary hospital in Buenos Aires, Argentina. In 2013, Becton Dickinson and Company (BD) licensed the development rights of the Odon device. In consequence, the trial was paused in January 2015 for BD to conduct preclinical studies [13–15] and develop a new prototype. 3) The third phase included multiparous and nulliparous women with follow-up until discharge at a public tertiary hospital in Pretoria, South Africa (2017). The new BD Odon device was planned to be applied in additional women at public hospitals in Argentina (2 hospitals), Kenya (1) and South Africa (4) for completion of the sample size, before the study was prematurely terminated. After the 49th case, the company decided to end this pilot study in favour of a randomized pivotal clinical trial to be conducted in Europe and India.

Women were invited to participate if they were between 18 and 35 years old, had no pre-existing health conditions and uncomplicated singleton pregnancies in the third trimester in Argentina or while in the hospital admitted for induction of labour in South Africa. Written informed consent was obtained before labour during antenatal care in Argentina or at the hospital after admission for childbirth in South Africa. Women were eligible for application of the Odon device during the second stage if the following conditions were met:

- fetus was alive and had a normal fetal heart rate as assessed by continuous electronic fetal monitoring;
- fully dilated cervix;
- ruptured membranes;
- any anterior occiput position;
- station level equivalent to 2 cm or more below the spines (station + 2 or lower).

Women were excluded if they did not confirm consent to participate in the study verbally before application of the device, or if any maternal or fetal complication arose during labour.

All women and their infants were followed until discharge and at 6-weeks postpartum. The first 30 mother/infant dyads recruited were also followed up to one year, as per protocol. No mother/infant dyad was lost to follow-up.

All the applications of the device were supervised, and assisted as required, by another obstetrician trained in the use of the device. A training plan for obstetricians applying the device was developed for implementation of the last phase of the study.

The Department of Reproductive Health and Research from the World Health Organization (WHO) was the sponsor and performed overall coordination of the study. A Data and Safety Monitoring Board (DSMB) and an expert committee independently reviewed the study progress and all cases. Based on these assessments, the study governing bodies supported continuing the series of studies necessary to evaluate feasibility, effectiveness and safety of the new BD Odon Device.

The study was approved by the WHO Research Ethics Review Committee; the Ethics Committee in Research of CEMIC and the National Drugs, Food, and Technology Administration of Argentina (ANMAT) in Argentina; and the The Research Ethics Committee, Faculty Health Sciences, University of Pretoria and the Medicines Control Council in South Africa. This study was registered in the Australian New Zealand Clinical Trials Registry (registration number ACTRN12613000141741).

### Intervention - the Odon device

**Fig. 1:**
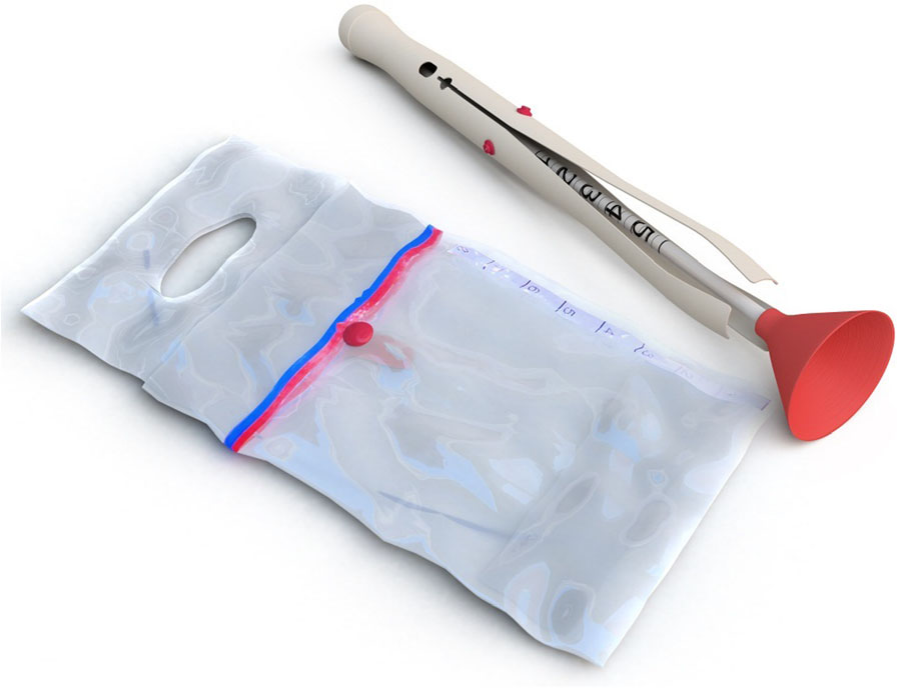
The Odon Device. Reproduced with permission of Schvartzman et al. Reproductive Health 2013, 10:33

**Fig. 2:**
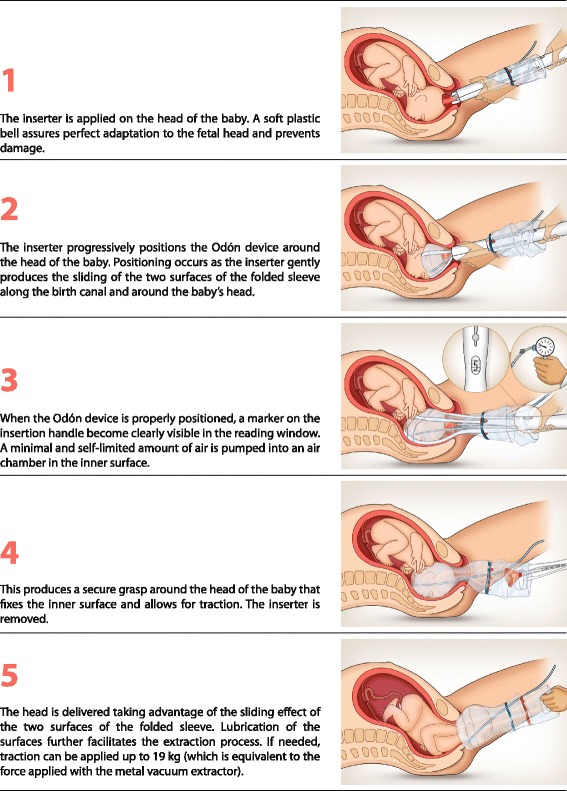
Visualization of the use of the Odon device. Reproduced with permission of Schvartzman et al. Reproductive Health 2013, 10:33

The Odon Device (Fig. 1) is made of two main components: a plastic sleeve and an inserter (or applicator). The sleeve is made of flexible polyethylene, with an internal fold in contact with the fetal head and the external fold in contact with the vaginal wall. The sleeve contains an air chamber (cuff) that is inflated around the fetal head by a manually operated bulb pump. The inserter consists of a handle with four pronged flexible spatulas that slide around the fetal head and help to position the sleeve. A plastic cup (plastic bell) at the tip of the inserter facilitates the application and protects the fetal head. The inserter has a progress indicator allowing the operator to check when the correct depth of insertion has been reached. The application technique of the Odon device is described in Fig. 2.

The devices used in Argentina were manufactured, and assembled by MDV (Muller, Dordoni, Visani) Srl in Buenos Aires, Argentina. The device used in South Africa was manufactured by BD in Singapore. During the study, design modifications, both to the inserter and the plastic sleeve, were introduced to improve usability, facilitate insertion and avoid loss of air pressure in the air cuff. Four slightly different prototypes of the device were used in Argentina. Device modifications were evaluated by the study DSMB and the Odon Device Research Group. These groups conferred that the modifications introduced potentially improved safety and usability and did not interfere with interpretation of the study results.

### Outcome measures

The list of safety and feasibility outcomes is available elsewhere [12]. Feasibility was assessed as successful application of the device defined as (1) reaching number 4 or 5 in the reading window of the inserter (Fig. 2, image 3), (2) successful inflation of the device without air leaks after the expulsion, and (3) successful expulsion of the fetal head with the plastic sleeve around the head (Fig. 2, image 5) after one-time application of the Odon device. Cases in which the plastic sleeve detached at the moment of crowning were reported as “crowning with Odon”. Insertion refers to the introduction and withdrawal of the inserter (Fig. 2, images 1 to 4).

Maternal and neonatal safety was assessed at labour, delivery and before discharge. Vaginal and cervical lacerations were evaluated by a systematic exploration of the birth canal and uterine cervix using vaginal spatulas after delivery. For the first 48 women/infant dyads the following safety outcomes were assessed at 6-weeks: perineal or vaginal haematoma, postpartum haemorrhage, infection, fever, blood transfusion, maternal or infant re-admission to hospital. For the first 30 dyads the following outcomes were assessed at the one-year follow-up: urinary incontinence, faecal/flat incontinence, perineal pain, dyspareunia, infant developmental impairment (assessed through maternal interview, contact with the paediatrician and review of medical record) and death.

### Sample size

A total sample size of 130 was estimated to measure potential maternal and infant complications with a reasonable precision, allowing detection rates of adverse maternal or infant outcomes between 2% (0.4%-6.1%) and 20% (13.5%-27.9%) with a 95% confidence interval width not larger than 15%. Details are published elsewhere [12]. The current study was terminated early in June 2017 in favour of a comparative trial. At that time, 48 women were recruited in Argentina and one woman in South Africa and results are presented in this manuscript.

### Statistical analysis

This is a descriptive analysis and no statistical inferences were done. Continuous variables are reported in means and ranges; categorical variables in percentages, with exact binomial 95% confidence intervals (CI) computed for successful application of the device and genital lacerations in the total sample.

## Results

Ninety women were invited to participate in the study during three study periods: between February 2011 and September 2012, March 2014 and January 2015, and May-June 2017. Exclusions included two women who did not fulfil eligibility criteria, 20 who did not provide written informed consent, and nine who were group B streptococcus (GBS) positive. At labour, one woman presented arrest of labour progress, one prolonged rupture of membranes, five had fetal complications and labour progressed too fast in one woman. Additionally, two women were excluded because their labours occurred during a study pause in recruitment in March 2014.

Forty-nine women were recruited, 30 multiparous women in the first phase, 18 in the second phase and one nulliparous woman in the third phase.

### Characteristics of the women

Table 1 shows selected characteristics of the women at the time of labour and delivery. The average maternal age was 31 years. The onset of labour was spontaneous in 40 cases and induced in eight. Augmentation of labour, with oxytocin or artificial rupture of membranes, was performed in 47 women. In Argentina, all women were under epidural anaesthesia.

**Table 1:** Characteristics of 49 women and their infants enrolled in the Odon device pilot study

	Mean or n (*N* = 49)	range or %
Maternal characteristics		
Maternal age (years)	31.2	(19-35)
Parity at eligibility		
Nulliparous	13	27%
Parity 1	21	43%
Parity 2 or more	15	30%
BMI at end of pregnancy	26.1	(22.7 – 40.8)
Labour and Delivery characteristics		
Spontaneous onset of labour	41	84%
Spontaneous rupture of membranes	14	29%
Augmentation of labour with oxytocin	39	76%
Vertex variety of position^a^		
Occiput anterior	36	73%
Left occiput anterior	10	20%
Right occiput anterior	3	6%
Epidural analgesia	48	98%

^a^One case was interpreted as anterior position at obstetrical examination but at delivery was occiput posterior position; *BMI* Body mass index

### Feasibility outcomes

Table 2 shows feasibility and safety outcomes in all women and by parity. The Odon device was successfully inserted in all women but three. The insertion process took on average 1 min and 27 s, with 34/49 of the insertions taking less than 1 min and 30 s (data not shown). Thirty-five out of 49 delivered successfully with the Odon device (71%, 95% CI 57-83%), with similar rates between nulliparous and multiparous women. In Argentina, successful Odon device deliveries was achieved more frequently with the improved and more advanced prototypes (22/27, 81%) than with the earlier ones (13/21, 61%) (data not shown).

**Table 2:** Feasibility of delivery with the Odon device in 49 women enrolled in the Odon device pilot study: All women and by parity

Indicators	Multiparous	Nulliparous	Total
	*N* = 36	*N* = 13	*N* = 49
	Mean or n (%)	Mean or n (%)	Mean or n (%)
Device application			
Fetal Station			
Station + 2/Hodge’s 3rd	22 (61%)	10 (77%)	32(65%)
Station + 3/Hodge’s 4th	14 (39%)	3 (23%)	17 (35%)
Vertex variety of position^a^			
Occiput anterior	26 (72%)	10^*^ (77%)	36 (73%)
Left or right occiput anterior	10 (28%)	3 (13%)	13 (27%)
Mean time of insertion (minutes:seconds)	01:39	00:50	01:27
Successful application of the Odon device			
Yes^b^	26 (72%)	9 (69%)	35 (71%)
No	10 (28%)	4 (31%)	14 (29%)
Spontaneous delivery, failed insertion	2	1	3
Spontaneous delivery, fetal descent with Odon	2	0	2
Spontaneous delivery, crowning with Odon^c^	4	2	6
Forceps	2	1	3
Reasons of non-successful delivery with Odon device			
Device was difficult to place	2	1	3
Device broke off	1	1	2
Device slipped off, air leaks^d^	5	1	6
Device slipped off, no apparent cause	2	1	3

^a^One case was interpreted as anterior position at obstetrical examination but at delivery was occiput posterior position

^b^Successful application of the device was defined as: (1) reaching number 4 or 5 in the reading window of the inserter, (2) successful inflation of the device without leaks after the expulsion, and (3) successful expulsion of the fetal head with the plastic sleeve around the fetal head after one-time application of the Odon device

^c^Plastic sleeve detached at the moment of crowning

^d^Five cases were caused by air leaks in the air cuffs, as documented by post-application examination of the cuffs, and one was caused by an air leak in the bulb pump

There were 14 non-successful applications according to the study predefined criteria. Two of failed insertions mentioned above were due to difficulties in positioning the device spatulas using the first prototype of the device, and the case in South Africa. The device slipped off in nine of the remaining cases and broke off (handle detached from the sleeve during traction) in two cases. Eleven of those women had a spontaneous delivery, and three had a forceps (two for maternal fatigue and one for fetal bradycardia).

### Maternal and infant outcomes

After delivery, perineal or vaginal tears were observed in two-thirds of the women (29/49, 59%, 95% CI 44-73%) (Table 3). Most of the tears occurred in nulliparous and were vaginal. There were no third or fourth degree perineal tears. Overall, 28 women received sutures. All four cervical tears occurred in the lateral sides of the cervix. One showed mild bleeding and all four were sutured following the local protocol. No other adverse maternal outcomes were reported.

**Table 3:** Maternal and neonatal outcomes during delivery and immediate postpartum (24-48 h) of 49 women enrolled in the Odon device pilot study: All women and by parity

Indicators	Multiparous	Nulliparous	Total
	*N* = 36	*N* = 13	*N* = 49
	Mean (range) or n (%)	Mean (range) or n (%)	Mean (range) or n (%)
Maternal outcomes			
Any vaginal or perineal tears^a^	18 (50%)	11 (85%)	29 (59%)
Any vaginal or perineal tears^a^ and/or episiotomy	23 (64%)	12 (92%)	35 (71%)
Vulvar tears	2 (5%)	0	2 (4%)
Vaginal lower half tears	9 (25%)	11 (85%)	20 (40%)
Vaginal upper half tears	0	0	0
Perineal 1st degree tear	8 (22%)	1 (8%)	9 (19%)
Perineal 2nd degree tears	1 (3%)	1 (8%)	2 (4%)
Perineal 3rd/4th degree tears	0	0	0
Episiotomy	7 (19%)	2 (15%)	9 (18%)
Cervical tears^b^	2 (5%)	2 (15%)	4 (8%)
Postpartum infection	1(3%)	1(8%)	2 (4%)
Infant outcomes			
Male	21 (58%)	9 (69%)	30 (61%)
Mean gestational age (weeks)	39.8 (37 - 41)	40.0 (38 - 41)	39.6 (37.0- 40.0)
Mean birth weight (grams)	3654 (2780 - 4560)	3488 (2880 - 4090)	3610 (2780 - 4560)
Apgar score at 5 min ≥ 7	36	13	49 (100%)
Caput succedaneum and moulding	3 (8%)	5 (38%)	8 (16%)
Cephalhematoma^c^	0	1 (8%)	1 (2%)
Admission to neonatal intensive care unit	1 (3%)	1 (8%)	2 (4%)

^a^Women may have more than one type of vaginal or perineal, excluding cervical tears

^b^Thirty-women had at least one type of vaginal, perineal or cervical tears

^c^One moderate

Before discharge from hospital, no serious maternal or neonatal adverse outcomes were reported. Two women received antibiotic treatment for foul smelling lochia with no fever, and one for urinary tract infection. One woman had hypoesthesia of the anterior region of the right leg thigh due to epidural anaesthesia. All perineal repairs healed normally. Two neonates were admitted to the neonatal intensive care unit for less than 7 days with respiratory distress for causes that were thought unrelated to the device. No other neonatal complications (jaundice, infection, and need of phototherapy, fetal or neonatal death) were reported.

No adverse outcomes were recorded in the six-week or one-year follow-up visits (for the first 30 women/infant dyads only). No unexpected adverse events were reported. No substantial differences were observed in safety outcomes among the five prototypes used (data not shown).

## Discussion

We evaluated feasibility and preliminary safety of application of the Odon device in 49 women with non-prolonged/non-complicated second stage of labour. The device was successfully inserted in 46 women, and successful delivery with the device was achieved in close to three-fourths of the women. Two-thirds of the women had vaginal, cervical or first/second degree perineal tears, but no third or fourth degree perineal tears were observed. No long-term maternal or neonatal adverse outcomes were observed.

Often new devices and procedures are introduced in medical practice without having been properly evaluated [16, 17], failing to provide adequate protection to patients and sufficient evidence on safety and to health care providers. However, the design of medical device studies requires specific approaches [18, 19]. This study was designed in phases to ensure safety by a periodic assessment of short- and long-term (one year) outcomes by an independent DSMB. For example, the design of the study included an evaluation of the first five cases and at one-year after delivery of the first 30 cases. As per protocol, upon conclusion of this follow-up period no further cases were recruited. This approach ensured that participants were not exposed to unnecessary risks and was also in line with requirements of the ethics committees. The design of the study in phases also allowed for introduction of modifications of the device during the study. The study DSMB and the Odon Device Research Group conferred that the modifications introduced potentially improved safety and usability and did not interfere with interpretation of the study results, as the principles of operation of the device did not change. While this is common in feasibility studies of new devices, and recognised by regulatory bodies [18], we acknowledge this may limit the interpretation of the results as a whole. Although the groups are small, no substantial differences were seen in terms of safety outcomes across the different prototypes used.

This study has several limitations. The study was carried out mainly at one hospital, and the majority of applications were performed by only two operators. If this facilitated acquisition of skills by the operators, feasibility still needs to be assessed with a larger number of operators and in different contexts. It was not possible to evaluate the level of discomfort and pain during application of the device, as all 48 enrolled women in Argentina were under epidural anaesthesia. It was planned to collect information on operator’s impressions of use during application of the device in the last phase of the study. However, this was not possible due to the early termination of the study. After the 49th case, the company decided to end this pilot study in favour of a randomized pivotal clinical study to be conducted in Europe and India. Ethical issues and consequences of premature discontinuation of studies have been extensively discussed in the literature [20–22].

It is difficult to compare the rates of vaginal/perineal tears in this study. While the overall rate of tears in the study (59%, *n* = 29/49) is comparable to rates associated with the hand held vacuum used in indicated cases: 45% of first or second degree tears; 68% of episiotomies; and 6% of third or fourth degree tears [8], which is the current available instrument associated with the lowest rate of perineal trauma. We acknowledge that the population in this study included women undergoing normal second stage of labour, while studies on the hand held vacuum [8] included a substantial proportion of women with prolonged second stage. Intact perineum in our sample compares to the 21 to 35% reported in the literature among low risk women (term, singleton, vertex presentation) [23]. It is notable that no intermediate or long-term adverse outcomes related to perineal trauma (e.g. perineal pain, incontinence, dyspareunia) were reported at the 6 week or 1 year follow-up.

We have observed four cervical tears in the lateral side of the cervix. The rate of clinically significant cervical tears after vaginal birth has been reported between 0.2 to 4.8% in different retrospective studies [24–27]. However, studies using routine colposcopy in consecutive cases of vaginal deliveries found much higher rates of any cervical injury, including erosion (79%), laceration (23% to 56%) and bruising (30%) without clinical signs [28–30]. One explanation of our findings is that the spatulas of the inserter may have caused harm by direct contact with the cervix. However, it is unlikely to be the only explanation, particularly with a fully dilated and effaced cervix and a fetus at station + 2 or lower. These findings may also be explained by observation bias. The fact that all women had a thorough vaginal examination may have prompted the diagnosis of an event that is underdiagnosed and underreported in routine practice [24].

At this stage, the increased risk of tears and other unknown risks cannot be ruled out. Also, it is not possible to know if women with indication for IVD could present higher rates of genital tears with Odon device compared to other instruments. Therefore, a fair assessment of the failures and complications commonly, such as birth canal tears, associated with IVD is not possible until a direct comparison between the Odon device and other existing instruments is conducted in a randomised clinical trial in the intended population. Further research is supported by results of preclinical studies showing that the Odon device was not associated with more perineal distension compared to forceps or vacuum [15]. Further improvements in the design of the device might also increase successful application in future research [31].

## Conclusions

Our results suggest that delivery using the Odon device is feasible. However, observed genital tears could be due to the device or the process of delivery and assessment bias.

If proof of concept has been demonstrated, effectiveness and safety of the device remains to be assessed in a randomised trial, as the device in this study was applied under non-emergency conditions, in women with normal progress of labour and no indication of IVD. Efficacy, safety and feasibility remain also to be assessed in different facility-settings and countries. A regular risk-benefit assessment will be needed in order to mitigate risks arising from this kind of study, and clear stopping rules shall be developed, including discontinuation for reasons not related to efficacy, safety, or feasibility. Safety outcomes, including risk of genital tears, including cervical tears, and pain during application need to be carefully assessed in future research.

## Abbreviations

BD: Becton Dickinson and Company
DSMB: Data and Safety Monitoring Board
GBS: Group B streptococcus
IVD: instrumental vaginal delivery
WHO: World Health Organization
